# TAS2R38 taste receptor gene and chronic rhinosinusitis: a bitter ending

**DOI:** 10.1186/2045-7022-5-S4-P3

**Published:** 2015-06-26

**Authors:** Giulia Montrasio, Stefania Gallo, Sarah Grossi, Giorgio Binelli, Raffaella Cinquetti, Daniel Simmen, Paolo Castelnuovo, Paola Campomenosi

**Affiliations:** 1 University of Insubria, ENT Department, Varese, Italy; 2 University of Insubria, Dipartimento di Biotecnologie e Scienze della Vita, Varese, Italy; 3 Klinik Hirslanden, ORL-Zentrum, Zurich, Switzerland

## Background

Chronic rhinosinusitis (CRS) is a frequent disease with a high social impact and multifactorial pathogenesis. Recently, single nucleotide polymorphisms (SNPs) within the *TAS2R38* gene were pointed at as possible contributors to the complex gene-environment interactions in CRS. This hypothesis was supported by *in vitro* evidence of the protective effect exerted by the functional bitter taste receptor T2R38 on sinonasal mucosa, due to its role in innate immunity. The purpose of this study was to confirm the proposed correlation between *TAS2R38* genotype and CRS comorbidities and to assess whether the presence of a particular allele can be considered a prognostic marker.

## Methods

Fifty-three CRS patients and thirty-nine healthy individuals were genotyped at the *TAS2R38* locus. CRS patients were treated by endoscopic sinus surgery and medical therapies and subdivided in “recalcitrant” and “healed”, depending on the clinical outcome, assessed by internationally accepted scoring systems. Chi-square analyses were used to assess the effect of genotype on CRS and CRS-related comorbidities.

## Results

The distribution of the different genotypes at the *TAS2R38* locus was not significantly different between recalcitrant CRS patients, healed CRS patients and controls (χ^2^_[10]_ = 2.75, p = 0,99). Besides, no associations were found between the different genotypes at the *TAS2R38* locus and CRS-related comorbidities.

## Conclusions

No association was found between *TAS2R38* alleles or genotypes and CRS, thus questioning its real contribution to CRS susceptibility. Further studies on larger cohorts are needed to verify these findings also *in vivo* and to shed light on the role of bitter taste receptors in CRS.

